# Salicylic Acid Release from Syndiotactic Polystyrene Staple Fibers

**DOI:** 10.3390/molecules28135095

**Published:** 2023-06-29

**Authors:** Verdiana Covelli, Antonietta Cozzolino, Paola Rizzo, Manuela Rodriquez, Vincenzo Vestuto, Alessia Bertamino, Christophe Daniel, Gaetano Guerra

**Affiliations:** 1 Department of Chemistry and Biology “A. Zambelli” and INSTM Research Unit, University of Salerno, Via Giovanni Paolo II 132, 84084 Fisciano, Italy; vcovelli@unisa.it (V.C.); acozzolino@unisa.it (A.C.); cdaniel@unisa.it (C.D.); gguerra@unisa.it (G.G.); 2 Department of Pharmacy, University of Salerno, Via Giovanni Paolo II 132, 84084 Fisciano, Italy; vvestuto@unisa.it (V.V.); abertamino@unisa.it (A.B.); 3 Department of Pharmacy, University of Napoli, Via Domenico Montesano 49, 80131 Napoli, Italy

**Keywords:** WAXD, polarized FTIR spectra, drug release, cellular activity

## Abstract

Films and fibers of syndiotactic polystyrene (sPS), being amorphous or exhibiting nanoporous crystalline (NC) or dense crystalline phases, were loaded with salicylic acid (SA), a relevant non-volatile antimicrobial molecule. In the first section of the paper, sPS/SA co-crystalline (CC) δ form is characterized, mainly by wide angle X-ray diffraction (WAXD) patterns and polarized Fourier transform infrared (FTIR) spectra. The formation of sPS/SA δ CC phases allows the preparation of sPS fibers even with a high content of the antibacterial guest, which is also retained after repeated washing procedures at 65 °C. A preparation procedure starting from amorphous fibers is particularly appropriate because involves a direct formation of the CC δ form and a simultaneous axial orientation. The possibility of tuning drug amount and release kinetics, by simply selecting suitable crystalline phases of a commercially available polymer, makes sPS fibers possibly useful for many applications. In particular, fibers with δ CC forms, which retain SA molecules in their crystalline phases, could be useful for antimicrobial textiles and fabrics. Fibers with the dense γ form which easily release SA molecules, because they are only included in their amorphous phases, could be used for promising SA-based preparations for antibacterial purposes in food processing and preservation and public health. Finally, using a cell-based assay system and antibacterial tests, we investigated the cellular activity, toxicity and antimicrobial properties of amorphous, δ CC forms and dense γ form of sPS fibers loaded with different contents of SA.

## 1. Introduction

Nanoporous crystalline (NC) phases, being suitable hosts of low-molecular-mass guest molecules, were described for two commercially relevant polymers: syndiotactic polystyrene [1,2,3,4,5,6] (sPS) and poly(2,6-dimethyl-1,4-phenylene) oxide [7,8,9,10] (PPO). Depending on their molecular structure, guest molecules can be slowly released from the polymer crystalline phases [11,12,13] or even stably hosted in the crystalline cavities [14,15,16], at least for temperatures lower than the polymer glass transition.

As for sPS, fibers exhibiting NC phases as well as host–guest co-crystalline (CC) phases, can be easily prepared, starting from amorphous fibers as obtained by traditional melt spinning plants [17,18,19,20]. In particular, in a recent paper [19], we found that guest-induced crystallization of melt-spun sPS fibers, also in the absence of mechanical stretching, can lead to high degrees of axial orientation of CC and derived NC phases. Hence, controlled sPS guest-induced crystallization can lead to fibers not only with relevant functionalities but also with improved mechanical properties. In principle, this makes possible simple preparation procedures leading to antibacterial fibers, textiles and fabrics, due to the occurrence of stable CC phases with bactericidal guest molecules. 

In this paper we explore different procedures leading to bactericide fibers, which host non-volatile antimicrobial guest molecules in the crystalline cavities of the NC δ form. In particular, the antimicrobial guest used in the present study is salicylic acid (SA) [21].

SA was reported to have antifungal and antibacterial effects, being able to induce morphological and physiological alterations in different compartments of fungal cells and bacteria [22,23,24]. Gram-positive and Gram-negative bacteria may colonize medical devices aggregating and growing in a peculiar form of microbial layer of cells attached to a substratum and nested in a matrix of extracellular polymeric materials, defined biofilms. The additional anti-biofilm-forming properties of SA make it a good candidate for many and various biomedical purposes, such as the prevention of biofilms on medical devices, which are typically the cause of chronic, nosocomial and medical device-related infections [25,26]. 

SA was also found as one of the major metabolites in cotton and, although present only in concentration of few ppm, it was suggested imparting delicate bactericide properties to cotton fabrics [27]. Antibacterial fabrics based on SA were already proposed in the literature, by bonding SA to the viscose fabrics [28] or to graphene oxide that in turn is used as additive of cotton [29]. 

In the first section of this paper, the sPS/SA CC δ form is characterized, mainly by wide angle X-ray diffraction (WAXD) patterns and Fourier transform infrared (FTIR) spectra of axially oriented films. In the second section, SA guest uptake and release for crystalline and CC sPS fibers, as obtained by guest-induced crystallization of amorphous melt spun fibers, are described.

To fully understand whether the obtained sPS/SA δ CC and γ forms could have an application in the field of health care as an antibacterial fiber, it was necessary to investigate the safety and lack of toxicity. To obtain these data, cell viability tests on keratinocyte cell lines were conducted. Finally, using bacterial strains of *Escherichia coli* and *Staphylococcus aureus*, the antibacterial properties of the sPS/SA fibers were tested.

## 2. Results and Discussion

### 2.1. Information on sPS/SA CC Form from Axially Oriented Films

A preliminary structural characterization study of a possible sPS/SA CC form was conducted on axially oriented sPS films with thickness of nearly 60 µm, for which high quality WAXD patterns, FTIR and polarized FTIR spectra can be easily achieved.

**Figure 1:**
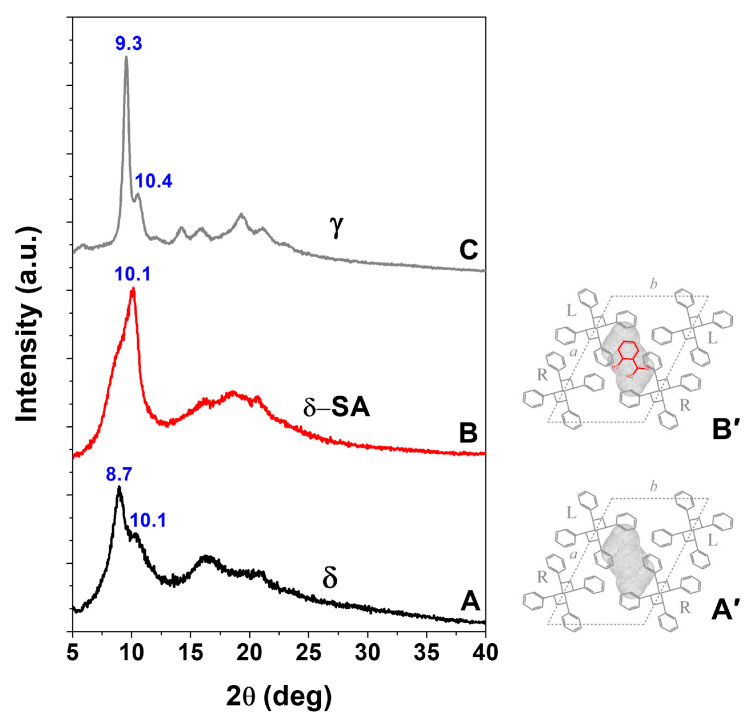
WAXD patterns, as collected by a powder diffractometer, of axially oriented sPS films exhibiting: (**A**) NC triclinic δ form; (**B**) CC triclinic δ form (SA content in the film of nearly 6 wt%); (**C**) γ form. Projections, along the axes of *s*(2/1)2 helices, of the crystal structures of: (**A′**) NC triclinic δ form; (**B′**) sPS/SA CC triclinic δ form. The SA guest orientation, preferentially perpendicular to the polymer chain axes (red guest molecule in **B′**), is deduced by polarized FTIR spectra reported in Figure 2.

WAXD patterns of a NC δ sPS film, as prepared by the procedure described in the experimental section, show the typical intense peak at 2θ = 8.7°, corresponding to the 010 reflection (Figure 1A) of triclinic δ phases [30]. The WAXD pattern of this NC δ sPS film, after immersion in a 24 wt% SA acetone solution, at 60 °C for 90 min, leading to a SA uptake close to 6 wt%, is shown in Figure 1B. As usually observed after guest sorption in the NC δ phase, the intensity of the 010 peak is decreased (here remaining present only as a shoulder) while a strong increase is observed for the diffraction peak at 2θ = 10.1°, corresponding to the $\bar{2}10$ reflection [30]. Hence, WAXD patterns of Figure 1A,B clearly indicate the inclusion of most SA molecules as guests of the NC triclinic δ form.

**Figure 2:**
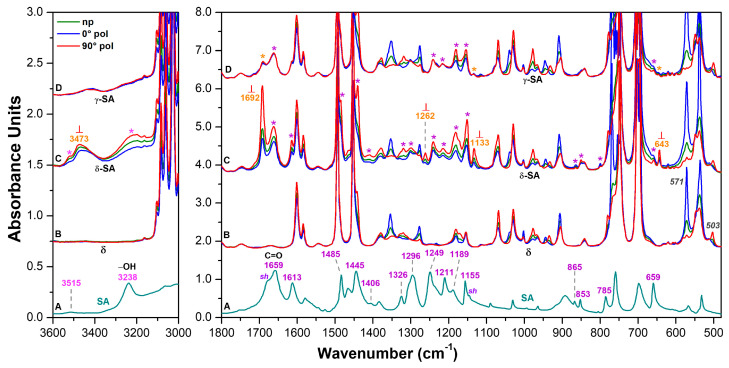
FTIR spectrum of SA KBr pellet (dark cyan line) in the spectral ranges 3600–3000 cm^−1^ and 1800–480 cm^−1^ is shown in (**A**). Unpolarized (green lines) and polarized FTIR spectra as taken with polarization plane parallel (blue lines) and perpendicular (red lines) to the film stretching direction, in the spectral ranges 3600–3000 cm^−1^ and 1800–480 cm^−1^, of axially oriented sPS films exhibiting: (**B**) NC triclinic δ form; (**C**) NC triclinic δ form after sorption of 6 wt% of SA; (**D**) γ form after sorption of 3 wt% of SA. Close to curves C, absorbance peaks of aggregated SA guest molecules are shown by asterisks while absorbance peaks of isolated SA guest molecules are labeled by orange wavenumbers.

FTIR spectrum of SA KBr pellet (dark cyan line) in the spectral ranges 3600–3000 cm^−1^ and 1800–480 cm^−1^ is shown in Figure 2A. Unpolarized (green lines) and polarized FTIR spectra of axially oriented sPS films, taken with polarization plane parallel (//, blue lines) and perpendicular (⊥, red lines) to the film stretching direction, are shown in Figure 2. Spectra of the NC triclinic δ form film (Figure 2B) show many dichroic peaks, corresponding to a high axial orientation factor (*S_δ_* = 0.8, calculated on the basis of dichroism of the 571 cm^−1^ vibrational peak). It is worth reminding that the orientation factor is equal to zero for random crystallite orientation, while it is equal to 1 for perfect parallel orientation of polymer chain axes of all crystallites.

FTIR spectra of the NC triclinic δ form after uptake of 6 wt% of SA (Figure 2C) also show many guest peaks, most of them being dichroic. This clearly confirms that most of SA guest molecules are included in the oriented NC phase, rather than in the poorly oriented amorphous phase. The spectra reported in Figure 2C show an additional relevant feature. In fact, beside SA peaks observed for FTIR spectra of solid SA (Figure 2A and second and fifth columns of Table 1) or of SA aqueous solutions (first column of Table 1) and labeled by asterisks in Figure 2C, new peaks appear at 3473 cm^−1^, 1692 cm^−1^, 1262 cm^−1^, 1133 cm^−1^ and 643 cm^−1^, being labeled by orange wavenumbers. These new peaks can be attributed to isolated SA molecules as segregated by crystalline cavities of the δ phase and are not present for spectra of solid SA as well as for SA aqueous solutions, due to the formation of hydrogen bonded SA aggregates. A strictly analogous phenomenon was recently observed for isolated benzoic acid guest molecules [16]. 

Particularly informative is the carbonyl region of the FTIR spectrum (Figure 2C) showing that the new stretching peak at 1692 cm^−1^, attributed to isolated SA molecules, is highly dichroic. The evaluated orientation factor of this peak is *S_SA,1692_* = −0.36, i.e., not far to the value corresponding to perfect perpendicular orientation (S = −0.5). This indicated that, in the sPS/SA CC triclinic δ form, SA molecules are placed in the crystalline cavities with their carbonyl group (and hence their phenyl ring) preferentially perpendicular to the polymer chain axis, as already observed for benzoic acid [16]. This preferential orientation of the SA guest molecules in the unit cell of the NC triclinic δ form [30] is schematically shown in Figure 1B′.

For the sake of comparison, spectra of a γ form sPS film after uptake of 3 wt% of SA are shown in Figure 2D. These spectra, showing highly dichroic polymer peaks, exhibit on the contrary all non-dichroic SA guest peaks. This confirms that, in films with the dense γ form, guest molecules can be absorbed only in their poorly oriented amorphous phase. It is worth adding that the presence of weak absorbance peaks at 3473 cm^−1^, 1692 cm^−1^, 1133 cm^−1^ and 643 cm^−1^ suggests the occurrence of a minor amount of isolated SA molecules, also in the amorphous phase. 

### 2.2. sPS Fibers with SA Guest

**Figure 3:**
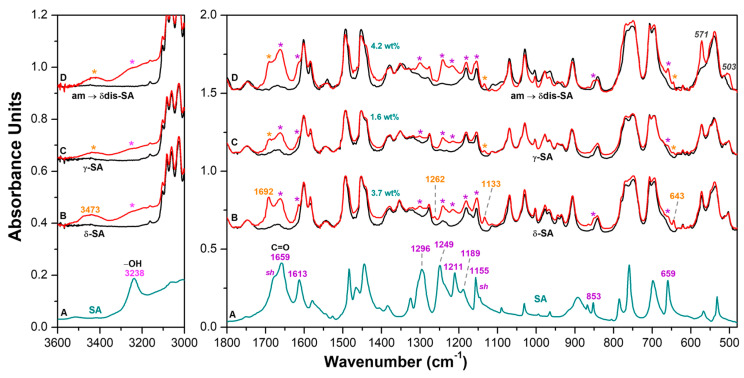
A FTIR spectrum of salicylic acid (dark cyan) in the spectral ranges 3600–3000 cm^−1^ and 1800–480 cm^−1^ is reported in (**A**). FTIR-DRIFT spectra in 3600–3000 cm^−1^ and 1800–480 cm^−1^ ranges of sPS fibers being: (**B**) semicrystalline with NC δ form; (**C**) semicrystalline with the dense γ form; (**D**) fully amorphous, which is crystallized by the guest sorption procedure. Black and red spectra correspond to fibers before and after SA sorption, respectively. Close to curves B, absorbance peaks of aggregated SA guest molecules are shown by asterisks while absorbance peaks of isolated SA guest molecules are labeled by orange wavenumbers.

**Figure 4:**
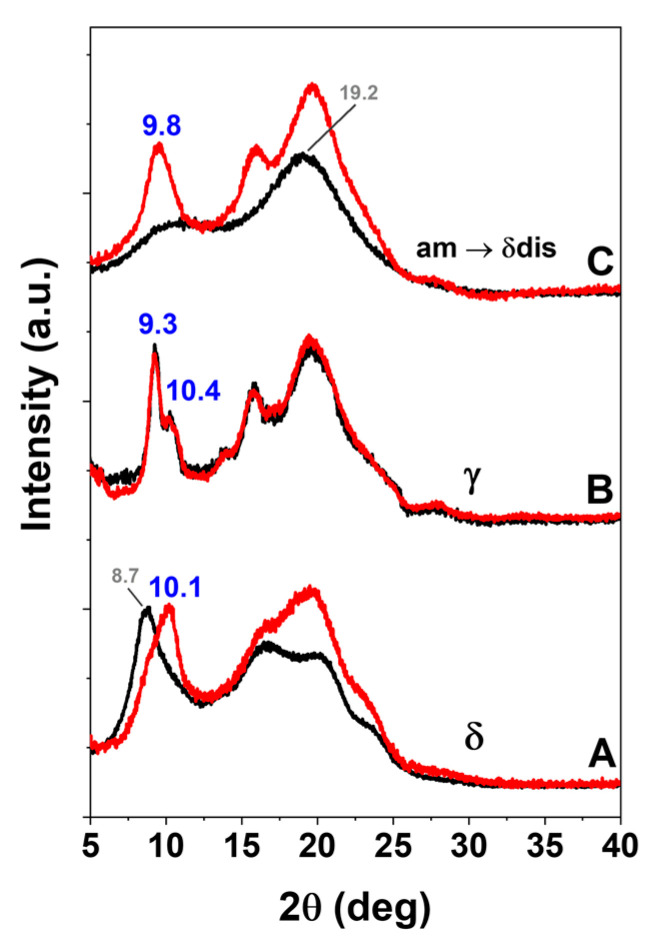
WAXD patterns of sPS staple fibers, exhibiting the NC δ form (**A**), the dense γ form (**B**), or being amorphous (**C**), before (black curves) and after treatment with SA/acetone solution (red curves). SA sorption leaves unaltered the diffraction pattern of the γ form staple (**B**), transforms the NC δ form into a CC δ form (**A**) and crystallizes the amorphous staple (**C**).

Amorphous sPS staple fibers as obtained by a melt spinning pilot plant as well as derived sPS staple fibers exhibiting the dense γ form or the NC δ form, were characterized by FTIR (DRIFT technique) and WAXD measurements (black curves in Figure 3 and Figure 4, respectively). These sPS staple fibers were loaded with SA antimicrobial molecules by soaking them in a 10 wt% SA/acetone solution at room temperature (≈20 °C) for 30 min. FTIR spectra and WAXD patterns of these sPS fibers with SA, after acetone desorption, are reported as red curves (Figure 3 and Figure 4).

FTIR-DRIFT spectra of Figure 3, as calibrated by thermogravimetric analysis (TGA), indicate that the SA uptake by the NC δ staple is definitely higher (3.7 wt%, Figure 3B) than for the γ form staple (1.6 wt%, Figure 3C). Particularly interesting is the FTIR spectrum of the amorphous staple, for which guest induced crystallization occurs. In fact, after soaking in SA/acetone solution, the sPS crystalline peaks appear, as for instance 571 cm^−1^ and 503 cm^−1^ peaks (Figure 3D), which correspond to vibrational modes of the TTGG regular conformational sequence [34,35,36]. The spectrum of Figure 3D also indicates that SA uptake is of 4.2 wt%, i.e., even higher than for the NC δ staple (Figure 3B). WAXD patterns of sPS staple fibers before and after SA sorption are shown in Figure 4. 

The WAXD pattern of NC δ form staple (black curve of Figure 4A) shows an intense equatorial 010 reflection at 2θ = 8.7°, typical of a triclinic δ phase [30]. After sorption of nearly 4 wt% of SA, the 010 reflection reduces its intensity and only appears as a shoulder of an intense $\bar{2}10$ reflection, at 2θ ≈ 10.1° (red curve of Figure 4A). This clearly indicates the formation of a triclinic sPS/SA CC phase [30]. The WAXD pattern of γ form staple (black curve of Figure 4B) shows typical narrow peaks at 2θ ≈ 9.3°, 2θ ≈ 10.4° and does not change as a consequence of SA sorption, which only occurs in its amorphous phase. The WAXD pattern of the amorphous staple (black curve of Figure 4C) only shows the typical broad amorphous halos, centered at 19.2°. This sample, after SA sorption from its acetone solution, crystallizes leading to a disordered δ CC phase, with typical diffraction peaks at 2θ ≈ 9.8°, 2θ ≈ 16.2° and 2θ ≈ 19.7° [19].

**Figure 5:**
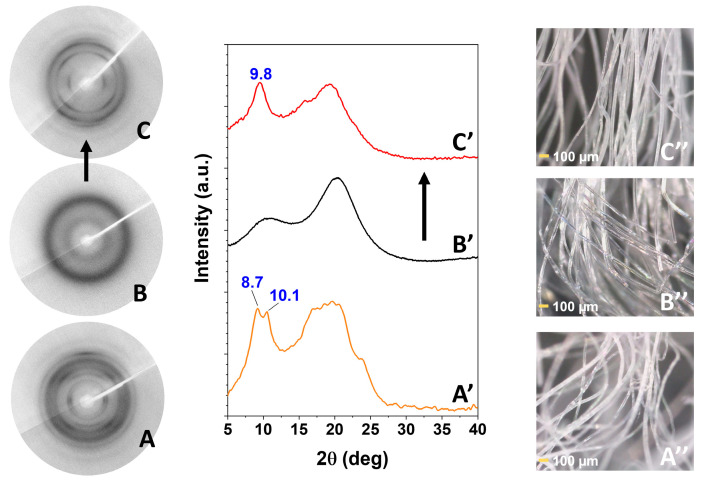
Two-dimensional-WAXD patterns (**A**–**C**) and corresponding equatorial profiles (**A′**–**C′**) of bundles of parallel staple fibers: (**A**,**A′**) triclinic sPS/SA CC δ form; (**B**,**B′**) amorphous; (**C**,**C′**) sPS/SA CC disordered δ form. Photographs of bundles of staple fibers presenting triclinic sPS/SA CC phase (**A″**) and of amorphous staple fibers before (**B″**) and after crystallization by SA sorption (**C″**) are also shown. The SA content in both CC sPS films is close to 4 wt%.

More precise information on the structure of the CC sPS/SA fibers of Figure 4A,C is obtained by two-dimensional (2D)-WAXD patterns of bundles of parallel staple fibers. A 2D-WAXD pattern of a CC δ form staple fiber is shown in Figure 5A. It is apparent that, as a consequence of the procedure of a guest (i.e., dichloromethane)-induced crystallization followed by SA sorption, crystalline arcs typical of the triclinic CC δ form appear in the main equatorial reflections at 2θ ≈ 8.7° and 10.1°, a meridional reflection at 2θ ≈ 22.7° and four main reflections on the first layer line at 2θ ≈ 13.4°, 16.4° and 2θ ≈ 20.1° and 23.4°. The presence of diffraction arcs rather than of diffraction rings indicates that the guest induced crystallization also leads to polymer crystallite orientation. The degree of axial orientation, evaluated as described in the experimental section, is high and close to *f_c_* ≈ 0.75.

Two-dimensional-WAXD patterns of amorphous staple fibers, before and after SA sorption, are also shown in Figure 5B,C, respectively. It is apparent that, as a consequence of the procedure of SA sorption, the circular broad amorphous halos are transformed in crystalline arcs: an equatorial reflection at 2θ ≈ 9.8°, a meridional reflection at 2θ ≈ 22.7° and two reflections on the first layer line at 2θ ≈ 16.2° and 2θ ≈ 19.7°, which are typical of the disordered δ form. Similar WAXD patterns corresponding to δ disordered crystalline phases were recently described in the literature, for instance by eugenol sorption by amorphous fibers [19]. The degree of axial orientation on sPS staple fibers crystallized from SA solution, evaluated as described in the experimental section, is close to *f_c_* ≈ 0.7. 

Finally, photographs of bundles of staple fibers presenting triclinic sPS/SA CC phase and of amorphous staple fibers before and after crystallization are also reported in Figure 5A**″**, 5B**″** and 5C**″**, respectively. It is clearly apparent that the transparent sPS amorphous staple fibers, as a consequence of crystallization, become opaque. 

### 2.3. SA Release and Retention in sPS Staple Fibers 

**Figure 6:**
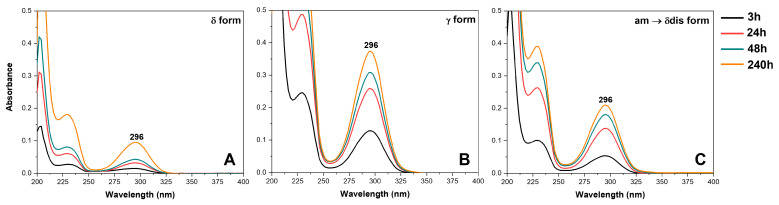
UV-vis spectra of SA released in PBS solution at selected times (3 h, 24 h, 48 h and 240 h) from different sPS staple fibers presenting: (**A**) CC δ form and SA content of 3.7 wt%; (**B**) γ form and SA content of 1.6 wt%; (**C**) CC disordered δ form and SA content of 4.2 wt%.

SA release from sPS staple fibers was monitored for 10 days (240 h) in phosphate buffered saline (PBS) solution, as described in the experimental section. Staple fibers showing dense γ and CC phases (δ and δ disordered) with SA amounts close to 1.6 wt%, 3.7 wt% and 4.2 wt% were immersed in a sealed vial containing PBS, under stirring at 100 rpm and at a temperature of 37 °C. Ultraviolet-visible (UV-vis) spectra of SA released after 3 h, 24 h, 48 h and 240 h are reported in Figure 6. It is apparent that a higher amount of SA was released in PBS solution by using γ staple fibers, irrespective of their lower SA content.

**Figure 7:**
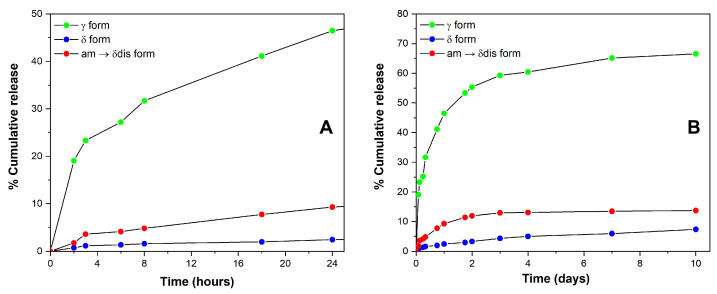
SA release (percent of the absorbed amount) in PBS aqueous solution at 37 °C: (**A**) from 0 to 24 h; (**B**) from 0 to 10 days, for different sPS fibers: (●) CC δ form and SA content of 3.7 wt%; (●) γ form and SA content of 1.6 wt%; (●) CC disordered δ form and SA content of 4.2 wt%.

Cumulative SA release, expressed in terms of weight percent, is shown in Figure 7. An immediate drug release profile (burst release) of 23.3%, 3.6% and 1.2% in the first 3 h of soaking is apparent for γ, CC δ disordered and CC δ fibers, respectively. The amount of SA released increases up to 46.5%, 9.3% and 2.5% after 24 h, and up to 55.3%, 11.9% and 3.3% after 48 h; then after 48–72 h (Figure 7B) the absorbance values reached almost a plateau and, after 10 days, the total amount released corresponds to 66.6%, 13.7% and 7.4% for γ, CC δ disordered and CC δ samples, respectively (Figure 7B). An initial burst release is commonly observed for polymer fibers or nanofibers containing drugs, it depends on many factors, for instance, the drug concentration on the fiber surface as well as polymer hydrophobicity and morphology [37,38,39,40,41]. In a recent work, Laezza et al. [24] reported a burst release for electrospun fibers obtained by combining poly-d,l-lactide (PDLLA), cellulose nanocrystals, chitosan and SA. In particular, they found a nearly linear sustained release in their system and a maximum SA release of about 50% [24]. The profile release herein shown for γ sPS staples (Figure 7, green circles) is comparable with that of the literature study. Hence, γ sPS staples could be considered suitable for topical administration of SA, which generally appears to be safe, as it shows no secondary effects such as gastric mucosal irritation and gastrolesivity [42,43]. SA, thanks to its lipophilicity, is able to enter the pilosebaceous units breaking the junctions between skin cells which leads to the flaking of the most superficial layers of the epidermis. Moreover, it quickly penetrates the inflamed lesions exerting its purifying, sebum-balancing and anti-inflammatory properties attenuating post-exfoliation irritations or those occurring in acne and psoriasis conditions [44,45].

Definitively slower is the SA release from CC staple fibers. For instance, after 8 h, the percentage of SA release is only 4.8% from CC δ disordered and 1.6% from CC δ staple fibers (Figure 7A). Of course, the slower release from CC staple fibers is due to the inclusion as isolated guest molecules in the crystalline cavities of the δ form. 

**Figure 8:**
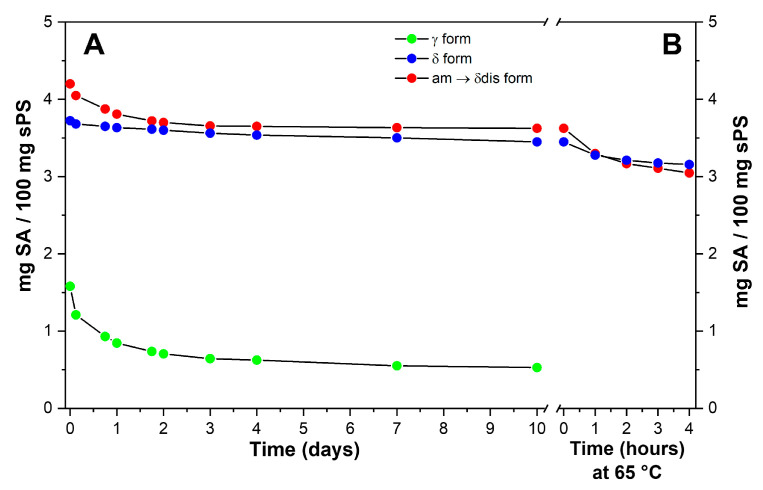
Salicylic acid percent content in the sPS fibers, in PBS aqueous solution at 37 °C (**A**) and after subsequent washing cycles of 1 h with water at 65 °C (**B**) from different sPS fibers: (●) CC δ form; (●) γ form; (●) CC disordered δ form.

The occurrence of large differences in SA guest retention by sPS staple fibers is shown in Figure 8A, where the content of SA in the sPS fibers, expressed as wt%, is reported versus the immersion time in the aqueous solution; γ staple fibers, which can absorb guest molecules only in their amorphous phases, are poorly loaded, but almost 60% of SA is released in 3 days (green circles and curve). Conversely, sPS staple fibers presenting CC phases (δ and δ disordered), which mainly absorb SA guest molecules in their crystalline phases, are loaded with a higher amount of SA and exhibit slower release kinetics, with active guest content becoming constant after the initial burst release. The effective retention of SA by sPS/SA CC fibers is confirmed by weight loss measurements on samples subjected to subsequent cycles of washing for 1 h with water at 65 °C (Figure 8B). For the sPS/SA CC δ form fibers, the weight content after two washing processes decreases from 3.4% to 3.2% and remains essentially constant for successive washing procedures. Hence, the results of Figure 8 indicate that sPS fibers exhibiting sPS/SA CC δ phases retain their antibacterial guest, even after repeated washing procedures.

### 2.4. Antimicrobial Activity of sPS Staple Fibers

**Figure 9:**
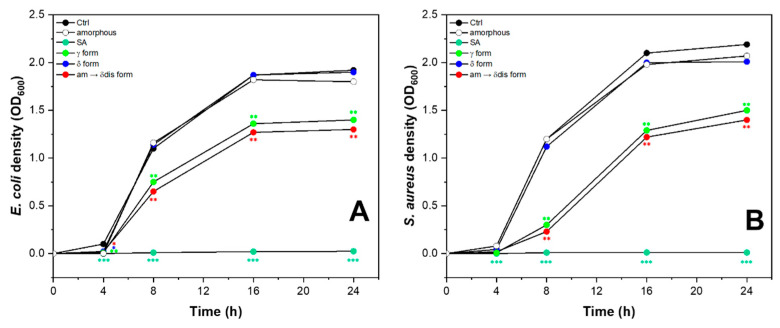
Antimicrobial activity of sPS staple fibers: (●) γ form; (●) CC δ form; (●) CC disordered δ form against *E. coli* (**A**) and *S*. *aureus* (**B**). SA (●) (2 mg/mL) was used as a positive control. Untreated bacteria (Ctrl, ●) and amorphous sPS staple fibers (○) were used as negative controls. The test was conducted through the exposure of sPS staple fibers in LB at 37 °C for 24 h measuring the bacterial density spectrophotometrically at 0, 4, 8, 16, 24 h. Data were compared using an analysis of variance test, and multiple comparisons were made with the Bonferroni’s test (* *p* < 0.05; ** *p* < 0.01; *** *p* < 0.001 vs. Ctrl).

In recent times, there has been a significant utilization of sPS fibers and nanomaterials in various strategies aimed at combating diseases and delivering drugs. These materials have proven to be effective in fighting bacteria and can be employed against both acute microbial diseases and bacterial strains that are resistant to multiple drugs [46,47]. In this regard, we investigated the antimicrobial activity of sPS staple fibers against strains of Gram-positive and Gram-negative bacteria. *Escherichia coli* is a widespread Gram-negative bacterium, known to cause common infections from contaminated water or food and *Staphylococcus aureus* is one of the most virulent pathogenic Gram-positive bacterium capable of giving rise to several diseases, some of them very serious, simply from contact with contaminated everyday items [48]. We selected *Escherichia coli* and *Staphylococcus aureus* strains in Luria–Bertani (LB) medium and tested the antimicrobial activity of different sPS staple fibers (amorphous, γ-SA 1.6 wt%, δ-SA 3.7 wt% and δ disordered-SA 4.2 wt%). Treatment with 2 mg/mL SA was used as a positive control. Untreated bacterial strains and amorphous sPS staple fibers were used as negative controls. Two of the sPS staple fibers analyzed, γ and CC δ disordered forms, inhibited both the growth of *E. coli* (Figure 9A) and *S. aureus* (Figure 9B). These interesting data are evidence of how sPS-SA-loaded staple fibers are able to inhibit the growth of various types of bacteria, including both Gram-positive and Gram-negative species. This means that these fibers can effectively impede the proliferation of a wide range of bacterial strains, regardless of their cell wall composition.

### 2.5. Skin Safety Evaluation of sPS Staple Fibers 

In order to determine the safety profile of our loaded sPS fibers, we measured the degree to which each fiber can induce a damage or death to cells. Cell viability and cytotoxicity assays are experimental in vitro toxicological methods used in basic research and drug development to screen chemical compounds to determine their safety profile and identify potentially harmful xenobiotics and toxicity in humans [49].

**Figure 10:**
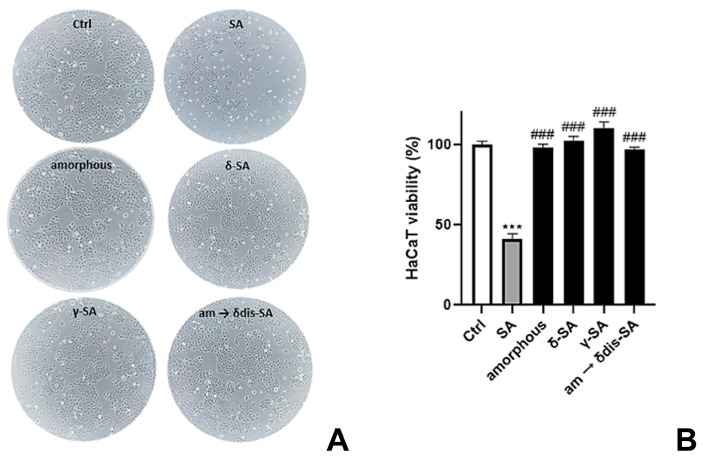
Cell safety evaluation of sPS fibers. (**A**) Images captured after sPS fibers were administered for 72 h. (**B**) Cell viability was performed using trypan blue assay. The trypan blue exclusion assay allows for a direct identification of live (unstained) and dead (stained) cells. SA (8 mg/mL) was used as positive control. *** denotes *p* < 0.001 vs. Ctrl. ### denotes *p* < 0.001 vs. SA.

Keratinocytes are the primary cells of the human epidermis providing the epidermal barrier function, playing a key role in the re-epithelization and inflammatory response of skin [50]. In particular, monolayer human immortalized non-tumorigenic keratinocyte cell line (HaCaT) are used for many skin model studies and applications, such as in vitro cellular toxicity analysis [51]. In vitro cytotoxicity of sPS fibers was evaluated by measuring the loss of cellular functions in HaCaT cell cultures via cell viability assay using trypan blue, a stain used to quantify live cells by labeling dead cells exclusively. In our tests, HaCaT cells were incubated with sPS fibers for 72 h followed by morphology evaluation and determination of cell mortality. As shown in Figure 10, the viability of cells treated without sPS fibers was defined as 100% (control group). 

SA (8 mg/mL) was used as positive control showing 41% of viability. The dose of SA used was chosen as a multiple of the maximum concentration of SA, considering the limiting case in which the 100% of the drug release form fibers is achieved. Only at these high doses of drug, administered neat in the absence of fibers, an interference in cell viability was noted. The relative cell viabilities of various groups treated with sPS fibers (δ-SA 3.7 wt%, γ-SA 1.6 wt%, am → δdis-SA 4.2 wt%) were always very high (>98.5%), showing no cytotoxicity of sPS fibers compared to SA used 20-fold concentrated. 

## 3. Materials and Methods

### 3.1. Materials

Syndiotactic polystyrene (Xarec 90ZC) was supplied by Idemitsu (Tokyo, Japan). Salicylic acid, solvents and reagents were purchased from Merck KGaA (Darmstadt, Germany) and used without further purification. Phosphate buffered saline solution (pH 7.4) was prepared using 0.1 M sodium hydrogen phosphate and 0.1 M sodium dihydrogen phosphate. The pH was measured with a GLP22 pH meter, CRISON instruments (Alella, Barcelona, Spain). 

### 3.2. Preparation of Films and Fibers

Amorphous sPS films were obtained by a blown extrusion process at 290 °C. Amorphous sPS films were axially stretched by a dynamometer INSTRON 4301 (Norwood, MA, USA) at 105 °C up to a draw ratio of 4.0. Axially oriented films presenting the NC δ phase were obtained by immersion of axially stretched amorphous films in dichloromethane (DCM) at room temperature for 3 h, followed by guest removal by acetonitrile (ACN) sorption for 1 h. Axially oriented films with the dense γ phase were obtained by annealing NC δ films at 170 °C for 1 h. The thickness of the axially oriented films was in the range of 50–70 μm.

In this study we have used staple fibers of sPS rather than continuous filaments because starting from staple fibers it is possible to achieve not only sPS yarns, but also mixed yarns with natural fibers (mainly with cotton), as described in detail in our patent [20].

Amorphous sPS staple fibers with a diameter of 35 µm were obtained by melt spinning with an extruding temperature of 290 °C. δ form staple fibers were obtained by immersion of amorphous staples in DCM at room temperature for 30 min, followed by guest removal through ACN sorption for 30 min. γ-form staple fibers were obtained by annealing of δ-form staple fibers at 170 °C for 1 h. Staple fibers showing a δ-disordered phase were obtained by direct immersion of amorphous sPS fibers in a SA-acetone solution. 

SA loading of sPS films and fibers was performed by immersion in a 24 wt% SA/acetone solution at 60 °C for 90 min and in a 10 wt% SA/acetone solution at room temperature for 30 min, respectively. In both cases, acetone removal was accelerated by placing the samples on a hot plate at ≈40 °C.

### 3.3. Structural Characterizations

FTIR spectra of films were collected at 2.0 cm^–1^ resolution in the wavenumber range 4000–400 cm^–1^ by a Vertex 70 Bruker spectrometer (Billerica, MA, USA), equipped with deuterated triglycine sulfate (DTGS) detector and a Ge/KBr beam splitter. Polarized infrared spectra of axially oriented films were recorded by using a SPECAC 12000 wire grid polarizer. FTIR spectra of fibers were collected in the diffuse reflectance infrared Fourier transform mode (DRIFT) by using an Easy Diff accessory benchmark from Pike Technologies. In order to reduce the noise 32 scans were signal averaged.

The intensity of FTIR guest peaks was used to evaluate the content of SA in films and fibers as calibrated by TGA. 

TGA measurements were performed with a TA Instruments Q500 thermal analysis system (New Castle, DE, USA), under N2 atmosphere in the temperature range of 25–500 °C and at a scanning rate of 10 °C/min.

The order parameter of the polymer crystalline phases (*S_p_*) as well as of SA guest molecules (*S_g_*), was calculated by the formula:

(1) $$S_{p/g}=\frac{R_{p/g}-1}{R_{p/g}+2}$$

where *R_p/g_* = A_//_/A_⊥_ is the dichroic ratio, and A_//_ and A_⊥_ are the measured absorbance for polarization plane parallel and perpendicular to the draw direction, respectively. This orientation factor is equal to zero for random crystallite orientation, while it is equal to +1 and −0.5 for orientation of polymer chain axes of the crystallites or of vibrational transition moment vector of guest molecules, being parallel and perpendicular to the stretching direction, respectively. 

X-ray diffraction analyses were recorded by an automatic Bruker D2 diffractometer (Billerica, MA, USA), while 2D-WAXD patterns were collected by a D8 QUEST Bruker diffractometer. In both cases, a nickel filtered CuKα radiation was used. 

The degree of crystallinity for all staple fibers was evaluated by using an automatic powder Bruker D2 diffractometer and it is in the range of 30–40%.

The degree of axial orientation (*f_c_*) was evaluated via the Herman’s orientation function:

(2) $$f_{c}=(3\;\overset{-}{{cos}^{2}x}\;-\;1)/2$$

where $\overset{-}{{cos}^{2}x}$ is the average cosine-squared value of the angle, *x*, between the *c* crystal axis and the fiber axis. When the *c*-axes of all crystallites are perfectly parallel to the fiber axis, *f_c_* is equal to 1 while for random crystallite orientation, *f_c_* is equal to 0. The degree of orientation of sPS fibers was evaluated by using azimuthal scans of the 002 reflection.

The images of sPS fibers were recorded by using a digital microscope by Dino-Lite Europe (Netherlands) with a magnification of 150× and a resolution of 1.3 Megapixel.

### 3.4. Drug Release Studies

**Figure 11:**
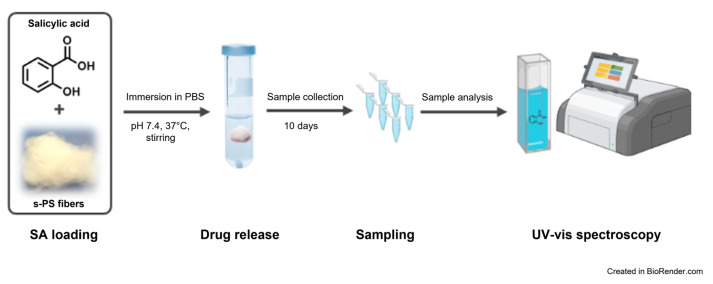
Workflow of the in vitro drug release studies.

In vitro drug release studies (Figure 11) were carried out by the total immersion method in PBS solution, an isotonic buffer solution commonly used in biological experiments, to reproduce the pH, osmolarity and ion concentrations of the human body. Indeed, a known quantity of the sample, approximately 27 mg of SA-loaded fibers presenting γ and CC δ and δ disordered phases were immersed in a closed vial containing 20 mL of PBS, under magnetic stirring at 100 rpm and a constant temperature of 37 (or 65) °C ± 2.0 °C. At selected time intervals (between 0 and 10 days), the release buffer solution was removed and replaced with an equal amount of fresh PBS to maintain the receptor phase starting volume. The SA release in solution was monitored by using UV-vis measurements, recorded on Thermo Scientific™ Multiskan™ GO spectrophotometer (Thermo Scientific, Waltham, MA, USA) in the range of 200–400 nm.

**Figure 12:**
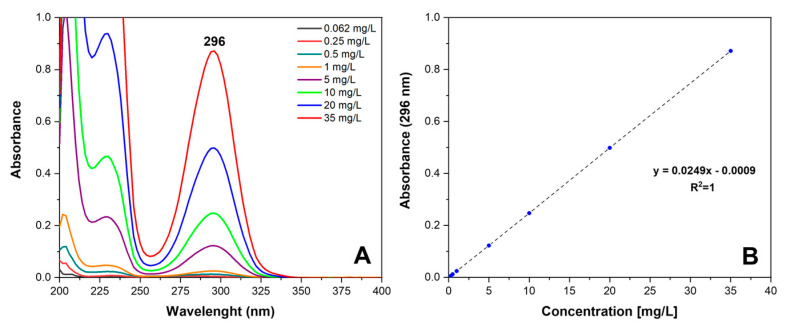
(**A**) UV-vis absorption spectra of salicylic acid in PBS solution in the concentration range 0.062–35 mg/L and (**B**) calibration curve.

A stock solution (70 mg/L) and serial dilutions ranging from 35 mg/L to 0.062 mg/L of SA in PBS were prepared, scanned in the UV range of 200–400 nm, and absorbance values were noted at λ_max_ = 296 nm (Figure 12A). A linear calibration curve (R^2^ = 1) (Figure 12B) was established and used to determine the SA concentration from the UV data. The cumulative release as a function of time was calculated according to Equation (3).

(3) $$\%\;Cumulative\;release=\frac{weight\;of\;the\;drug\;released(mg)}{weight\;of\;the\;drug\;in\;the\;fiber(mg)}\times 100\;(\%)$$

### 3.5. Investigation of Antimicrobial Activity

*E. coli* (ATCC 15597) and *S. aureus* (ATCC 23235) strains were purchased from ATCC (Manassas, VA, USA). Briefly, bacterial cultures for treatments were prepared from a single colony picked from a freshly streaked LB Agar Miller plate and inoculated into 5 mL of LB medium and grown to reach logarithmic phase (8 h, 37 °C, 180 rpm). After, an aliquot of the starter culture was diluted 1/500 into 150 mL of LB medium and grown to saturation in overnight culture (16 h, 37 °C, 180 rpm).

For the treatments, the cells were inoculated at a density of 5 × 10^4^ cells/mL in fresh LB medium mixed with sPS fibers (amorphous, γ-SA 1.6 wt%, δ-SA 3.7 wt% and δ disordered-SA 4.2 wt% as reported before) and incubated under shaking conditions at 37 °C for 24 h, measuring OD_600_ of suspension cultures at 0 h, 4 h, 8 h, 16 h and 24 h using a spectrophotometer (Multiskan Go, Thermo Scientific, Waltham, MA, USA). The experiments were repeated twice.

Statistical analysis was performed using an analysis of variance test, and multiple comparisons were made with the Bonferroni’s test with GraphPad Prism 8.0 software (San Diego, CA, USA). Significance was assumed at *p* < 0.05. Data are reported as mean ± SD of results from two independent experiments.

### 3.6. Cell Cultures and Determination of Cell Viability

HaCaT cell line, a kind gift of Prof. Ornella Moltedo (Department of Pharmacy, University of Salerno, Fisciano, Salerno, Italy), was cultured in Dulbecco’s Modified Eagle Medium (DMEM, 4500 mg/mL glucose) supplemented with 10% (*v*/*v*) fetal bovine serum, 2 mM l-glutamine, 100 U/mL penicillin and 0.1 mg/mL streptomycin. Cells were routinely grown in culture dishes (Corning, Corning, New York, NY, USA) in an environment containing 5% CO_2_ at 37 °C and passaged at confluence using a solution of 0.25% trypsin and 0.1% EDTA. In each experiment, cells were placed in a fresh medium, cultured in the presence of sPS fibers and followed for the analyses. Experiments were performed in duplicate.

HaCaT cell line (5 × 10^5^ cells/well) was seeded in 6-well plates and treated with sPS fibers (δ-SA 3.7 wt%, γ-SA 1.6 wt%, am → δdis-SA 4.2 wt%, as reported before) for 72 h. Cell mortality was performed by using trypan blue assay. Cell viability was expressed as a percentage relative to the untreated cells cultured in medium and set to 100% of viability. Data were reported using GraphPad Prism 8.0 software. Images were captured using LEICA ICC50 HD light microscope (10×) (Leica Microsystems, Wetzlar, Germany). 

Statistical analysis: data are reported as mean ± SD of results from two independent experiments. Statistical analysis was performed using an analysis of variance test, and multiple comparisons were made with the Bonferroni’s test with GraphPad Prism 8.0 software (San Diego, CA, USA). Significance was assumed at *p* < 0.05.

## 4. Conclusions

Structural studies, mainly based on polarized FTIR spectra and WAXD patterns on axially oriented sPS films, have clearly shown the formation of a sPS/SA δ CC form, with isolated SA guest molecules being preferentially perpendicular to the axes of the crystalline *s*(2/1)2 helices.

The occurrence of a sPS/SA δ CC form allows preparation of sPS staple fibers even with a high content of the antiseptic guest (at least up to 4 wt%), which is retained by the fiber even after long term immersion in water at room temperature as well as after repeated washing procedures at 65 °C. sPS fibers exhibiting sPS/SA δ CC phases can be prepared by room temperature treatments with SA solutions in acetone not only of sPS fibers exhibiting the δ NC form but also of sPS amorphous fibers (as directly obtained by melt spinning processes). A detailed WAXD characterization study on bundles of parallel staple fibers shows that procedures starting from NC δ and amorphous samples lead to triclinic δ CC and disordered δ CC phases, respectively. The procedure starting from amorphous fibers is particularly appropriate because not only involves a direct formation of the CC phase but also a simultaneous axial orientation (with related improvement of mechanical behavior). 

Lower amounts of SA can be also loaded in sPS staple fibers exhibiting dense crystalline phases, which are able to absorb guest molecules only in their amorphous phases. For instance, drug release studies performed on SA loaded (1.6 wt%) γ form staple fibers have shown a burst drug release in the first 8 h of nearly 32% that goes up to nearly 60% after 72 h. Hence, sPS staples exhibiting the dense γ phase could be considered for administration by topical application of SA.

In summary, with this study we showed the possibility of tuning both loaded drug amount as well as drug release kinetics, by simply selecting suitable crystalline phases of a commercial thermoplastic polymer, making sPS fibers possibly useful for many applications. For the first time we also demonstrated that native sPS fibers and SA-loaded-fibers exhibit a non-toxic profile in in vitro skin cell models, warranting use in the field of health care. Additionally, antibacterial tests against *Escherichia coli* and *Staphylococcus aureus* strains evidenced a significant reduction in bacterial growth, making sPS fibers loaded with different SA contents useful for antimicrobial properties.

In particular, fibers with sPS/SA δ CC phases could be useful for preparation of textiles and fabrics, which require antimicrobial guest retention. Fibers with sPS γ form could be used for cosmeceutical and medical patches that, on the contrary, require antimicrobial guest release (for instance, for delayed action on the skin). 

## Figures and Tables

**Table 1:** FTIR peaks of SA as observed in aqueous solution, [31] in KBr pellet, [32] and for SA guest molecules in axially stretched sPS δ-form films. The third column collects the literature information on vibrational assignments [31,32,33]. FTIR peaks of SA as collected in the present paper are reported in the last two columns.

SA in Aqueous Solution [31]	SA in KBr Pellet [32,33]	Vibrational Assignment [32,33]	SA in CC δ sPS	SA in KBr Pellet (Present Work)
			**643** ⊥	
	659 [32]	β OCO + β OCC + τ CCCC [32]	659 //_w_	659
	801_calc_/805_exp_ [33]	γ (COOH) + γ (OH)_mon_ + γ (CH)_rings_ [33]	800 //	785
852	852 [32]		851 ⊥	853
861			864 //_w_	865
			**1133** ⊥	1145 sh
1148	1155 [32]	ν CC + β HOC + β HCC [32]	1152 ⊥	1155
	1191 [32]	β HOC + β CCH [32]	1181 ⊥	1189
			1214 ⊥	1211
1246	1248 [32]	β HOC [32]	1241 ⊥	1249
			**1262** ⊥	
1326	1324 [32]	ν CC + β HOC + β HCC [32]	1319 ⊥_w_	1326
			1409 ⊥_w_	1406
	1441 [32]	ν CC + β HCC [32]	1441 ⊥_w_	1445
1488			1485 ⊥_w_	1485
1610	1611 [32]	ν CC [32]	1614 ⊥	1613
1660	1667_calc_/1658_exp_ [33]	ν (C=O) + ν (CC)_rings_ [33]	1661 ⊥	1659
			**1692** ⊥	1679 sh
	3239_exp_ [33]	ν (OH)_phen_ [33]	3219 ⊥	3238
			**3473** ⊥	
	3505 [32]	ν OH [32]	3522 ⊥	3515

γ: out-of-plane bending; β: in-plane bending; τ: torsion; ν: stretching. Note: new experimental vibrational peaks in sPS samples are labeled as bold red wavenumbers. Spectra taken with polarization plane parallel (//) and perpendicular (⊥) to the film stretching direction. w = weak absorbance peaks

## Data Availability

Data unavailable.

## Sample Availability

Samples of the compounds are not available from the authors upon request.
